# Assessment of Short-Term Engraftment Potential of *Ex Vivo* Expanded Hematopoietic Stem Cells Using Normal Fetal Mouse in Utero Transplantation Model

**DOI:** 10.22074/cellj.2019.6006

**Published:** 2019-06-15

**Authors:** Morteza Zarrabi, Elaheh Afzal, Mohammad Hassan Asghari, Marzieh Ebrahimi

**Affiliations:** 1 Department of Stem Cells and Developmental Biology, Cell Science Research Center, Royan Institute for Stem Cell Biology and Technology, ACECR, Tehran, Iran; 2 Royan Stem Cell Technology Company, Cord Blood Bank, Tehran, Iran; 3 Animal Core Facility, Reproductive Biomedicine Research Center, Royan Institute for Animal Biotechnology, ACECR, Tehran, Iran

**Keywords:** Chimerism, Cord Blood Stem Cell Transplantation, Hematopoietic Stem Cells

## Abstract

**Objective:**

*Ex vivo* expansion is a promising strategy to overcome the low number of human umbilical cord blood hematopoietic stem cells (hUCB-HSCs). Although based on the obtained results in unnatural physiological condition of irradiated genetically immune-deficient mouse models, there has always been concern that the expanded cells have less engraftment potential. The purpose of this study was to investigate effect of common *ex vivo* expansion method on engraftment potential of hUCB-mononuclear cells (MNCs), using normal fetal mouse, as a model with more similarity to human physiological conditions.

**Materials and Methods:**

In this experimental study, briefly, isolated hUCB-MNCs were cultured in common expansion medium containing stem cell factor, Flt3 ligand and thrombopoietin. The unexpanded and expanded cells were transplanted to the fetal mice on gestational days of 11.5-13.5. After administration of human hematopoiesis growth factors (hHGFs), presence of human CD45^+^ cells, in the peripheral blood of recipients, was assessed at various time points after transplantation.

**Results:**

The expanded MNCs showed 32-fold increase in the expression of CD34+38- phenotype and about 3-fold higher clonogenic potential as compared to the uncultured cells. Four weeks after transplantation, 73% (19/26) of expanded-cell recipients and 35% (7/20) of unexpanded-cell recipients were found to be successfully engrafted with human CD45^+^ cells. The engraftment level of expanded MNCs was significantly (1.8-fold) higher than unexpanded cells. After hHGFs administration, the level was increased to 3.2, 3.8 and 2.6-fold at respectively 8, 12, and 16 weeks of post transplantation. The increased expression of CXCR4 protein in expanded MNCs is a likely explanation for the present findings.

**Conclusion:**

The presented data showed that expanded MNCs compared to unexpended cells are capable of more rapid and higher short-term engraftment in normal fetal mouse. It could also be suggested that in utero transplantation (IUT) of normal fetal mice could be an appropriate substitute for NOD/SCID mice in xenotransplantation studies.

## Introduction

Hematopoietic stem cells (HSCs) are multi-potent progenitor cells having the ability of both multi-lineage differentiation and long-term self-renewal (1). A single HSC can repopulate the entire hematopoietic system. Therefore, over the last six decades, HSC transplantation has been widely used to treat various hematological disorders and malignancies (2, 3). Although human umbilical cord blood (hUCB) is an invaluable source of HSCs for cell therapy, the limited cell dosage of HSCs in a cord blood (CB) unit results in delayed post-transplant hematologic recovery. So, CB transplantation has mostly been limited to pediatric or adults with low body weight (4). Although, *ex vivo* expansion is the most promising strategy for overcoming the limited number of hUCBHSCs, there is always a concern that expanded HSCs have less potency for hematopoiesis and engraftment compared to unexpanded ones (5). Therefore, the efficacy and safety of expanded cells must clearly be evaluated before use in clinic.

So far, significant advances in our understanding about the *in vivo* functions of human HSCs have resulted from development of severely immune-deficient models such as non-obese diabetic/severe combined immunodeficiency (NOD/SCID), NOD/SCID/gamma (NSG) and nude mice (6, 7). Most of these immune-deficient mice have short lifespan with difficult breeding performance and their husbandry requires specific pathogen-free (SPF) environment (8). Moreover, due to the lack of appropriate human immune cells traffic as well as lack of completely normal physiological environment, the derived data from such mice cannot be considered as a precise reflection from the real situation in our body. On the other hand, in these models, the engraftment level of human adult stem cells in the most organs other than hematopoietic system is very low (9). Because of the drawbacks associated with abnormal physiological condition of the models, there is considerable skepticism about the obtained results from such mice. So, there is still a need to find an animal model with more similarity to the *in vivo* environment of human body which can also be accessible for all laboratories, especially for whom with limited animal facilities.

In the last two decades, for allo- or xeno-transplantation studies, in utero transplantation (IUT) model of various animal fetuses such as mice (10), dogs (11), pigs (12), monkeys (13) and sheep (14) have alternatively been used to the genetically immune-deficient mice. For example, using a sheep IUT assay, it has been shown that a non-integrating and non-replicating Sendai virus vector expressing *HoxB4* gene can efficiently enhance the *ex vivo* expansion of hUCB-CD34^+^ cells (15). Furthermore, it has been demonstrated that treatment of pregnant sheep by busulfan (a myeloablative agent), 6 days before IUT, could improve engraftment level of human cells (16). In IUT model, before immunologic maturity of the fetus (when chimerism and donor-specific immune tolerance can be created), allo- or xeno-geneic cells intrauterine transplantation is performed. Since there is no need to use myeloablative drugs or irradiation, immature preimmune fetuses of animals could be an ideal, inexpensive and powerful models for biomedical research. In addition to its use for study basic questions in developmental and stem cell biological approaches, IUT of foreign progenitor or stem cells to the unborn fetus has potential to treat and ideally cure a number of congenital hematologic and nonhematologic disorders, prior to birth (17-19).

Although large animals allow long-term and higher level of donor cell engraftment, they do require more cumbersome facilities for maintenance and examination. Therefore, it seems that small rodents such as mouse are more useful IUT models, by supplying a larger number of animals and limited facilities. In our knowledge, a few studies used fetal mouse to investigate the *in vivo* behavior of hUCB-HSCs. In this study, we used the fetal mouse IUT model to assess the effect of common *ex vivo* expansion method on the engraftment potential of hUCBMNCs.

## Materials and Methods

### Preparation of human donor cells

In this experimental study, cells were obtained from UCB samples of mothers who consented according to guidelines established by the institutional human research Ethics review Committee of Royan Institute and Royan Stem Cell Technology Company (www.rsct.ir), Iran. Animal experiment was approved by the Institutional Animal Care and Committee of Royan Institute (IR. ACECR.ROYAN.REC.1394.175).

At first, 6% hydroxyethyl starch was used to sediment CB erythrocytes. Low-density MNCs were separated by lymphoprepTM (Stemcell Technology Inc., Canada) density-gradient centrifugation at 22˚C, 800 g for 30 minutes. MNCs (106/well) were cultured for 10 days in the StemSpanTM medium (Stemcell Technology Inc., Canada) containing the following human recombinant cytokines all obtained from R&D Systems (USA): stem cell factor (SCF) 100 ng/ml, Fms-like tyrosine kinase 3 ligand (Flt3L) 100 ng/ml and thrombopoietin (TPO) 50 ng/ml. Freshly isolated and expanded UCB-MNCs were stained with the following antibodies against human cells: CD34-FITC, CD38-PerCP and CXCR4-PE. Appropriate isotype controls were also used to delete non-specific background signals. All of the antibodies were purchased from BD Pharmingen™ (USA) except CXCR4 which was obtained from BioLegend, USA. After staining, the cells were analyzed on Partec flow-cytometer (Germany) and the data were analyzed using FlowMax software. Before transplantation, the cells were labeled by PKH26 cell tracking dye (Sigma, USA) according to manufacturer’s instruction and they were suspended in modified Dulbecco media containing 10% fetal bovine serum (FBS), for future use.

### Colony-forming cell assay

Briefly, 2000 MNCs were suspended in 0.3 ml IMDM+2% FBS and added to a 3 ml MethoCult™ (Stem Cell Technologies, Canada) tube for a duplicate assay. After 12-14 days of culture, each plate was scored for granulocyte macrophage colony-forming unit (CFUGM), burst forming unit-erythroid (BFU-E), as well as granulocyte, erythroid, macrophage and megakaryocyte colony-forming unit (CFU-GEMM).

### Transwell migration assay

The migration assay was performed using 24-well transwell plates (Corning Costar, USA) with 5 μm pore filters. The upper chambers were loaded with freshly isolated or 10 days expanded MNCs (106 cells) in 100 μl medium, while StemSpan medium and 100 ng/ml stromal cell derived factor-1 (SDF-1, R&D Systems) were placed into the lower chamber. After 4 hours incubation at 37˚C, the migrated cells to the lower side of the filter were collected and counted.

### In utero stem cell transplantation

**Fig.1:**
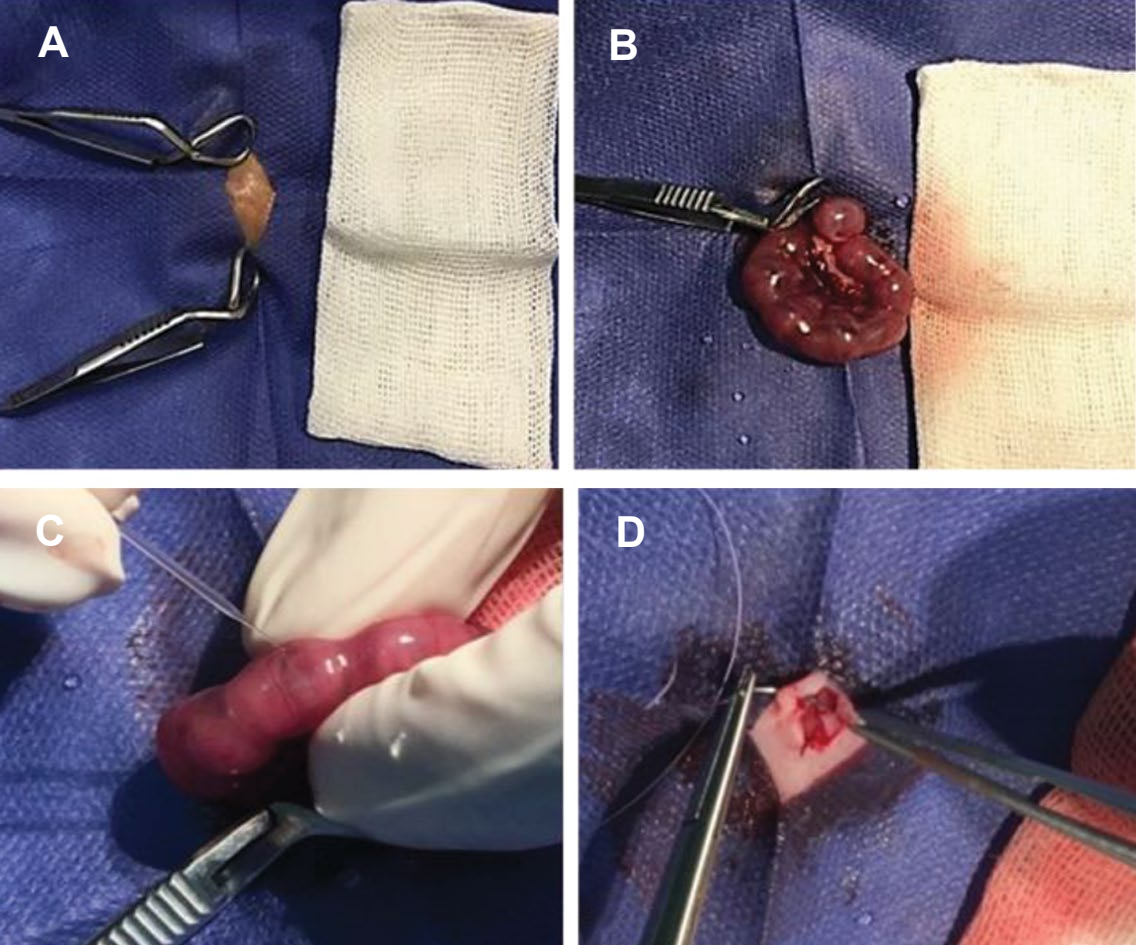
In utero transplantation of hUCB-MNCs using handmade glass micropipettes. **A. **Hair removal and sterilization of the surgical site, **B.** The uterine horns were exteriorized, **C. **2-3×106 non-cultured hUCB-MNC or their entire progeny, following 10 days expansion, were injected intraperitoneally to each recipient, and **D**. The uterine horns were returned to the abdominal cavity and the incision was closed. hUCB-MNCs; Human umbilical cord blood-mononuclear cells.

NMRI pregnant mice were supplied by center of experimental animals of Royan Institute (Iran). Briefly, on embryonic days E11.5- E13.5, the pregnant mice were anesthetized by isoflurane inhalation and the uterine horns were exteriorized. Using handmade glass micropipettes with 70 μm diameters, each embryo was intraperitoneally injected with 50 μl phosphate buffer saline (PBS) containing 2-3×10^6^ hUCB-MNCs or their entire progeny following 10 days expansion. Sham group received only 50 μl PBS. The uterine horns were returned to the abdominal cavity and the incision was closed with absorbable suture (Fig .1). The mothers were left undisturbed without bedding changes until the pups were 3 weeks old.

### Growth factor treatment

Evaluation of chimerism was performed monthly up until age of 4 months. Considering the average weight of a mouse at the age of 4, 8, 12 and 16 weeks of age (which is respectively around 10-12, 26-35, 32-48, and 35-50 g), the recipients were treated with subcutaneous injections of human recombinant proteins all of which were obtained from R<D system: SCF (4 ng/g), Interleukin-3 (IL-3, 4 ng/g) and granulocyte-colony stimulating factor (G-CSF, 50 ng/g) for 3 times a week beginning at 3 weeks of age.

### Immunostaining analyses of donor mononuclear cell

Following birth, several mice, transplanted with PKH26 labeled-MNCs, were sacrificed and frozen sections were prepared on albumin-coated slides from formaldehydefixed, optimal cutting temperature compound (OCT)- embedded liver and spleen of newborn mice. The prepared slides were subjected to detect PKH26-labeled human cells using a fluorescence Nikon microscope.

Moreover, bone marrow cells were aspirated from the tibia/femur and fixed on positively charged slides with ice-cold acetone. The cells were then incubated with Anti- Human Nuclear Antigen antibody (HNA, AbCam, UK) in a humidified chamber overnight at 4˚C, processing with secondary antibodies for one hour at room temperature in dark. The HNA immunostaining were observed using a fluorescence Nikon microscope.

To assess chimerism, 4 weeks after birth, 2-10 μl of peripheral blood was collected in heparinized tubes via the tail tip excision and partial amputation of the tail. The red blood cells were lysed with ammonium chloride lysis buffer and washed with PBS. The cells were then blocked with 1% bovine serum albumin (BSA) and stained with anti-Human CD45/34 or anti-Human Isotype Control (both from BD, USA) for 30 minutes at 4˚C. After staining, at least 10^5^ cells were analyzed on Partec flow-cytometer and the data were analyzed using FlowMax software. Engraftment was defined as detection of 0.2% or more human CD45 cells.

### Statistical analysis

All data are expressed as the mean ± SD. Significance of the differences between groups was determined using two-tailed Student’s t test assuming unequal variances. The level of significance was set at P<0.05. The statistical analysis was carried out using SPSS version 16 (SPSS Inc., Chicago, Il, USA).

## Results

### Increased *in vitro* proliferation and differentiation potential of the expanded human umbilical cord blood-mononuclear cells

**Fig.2:**
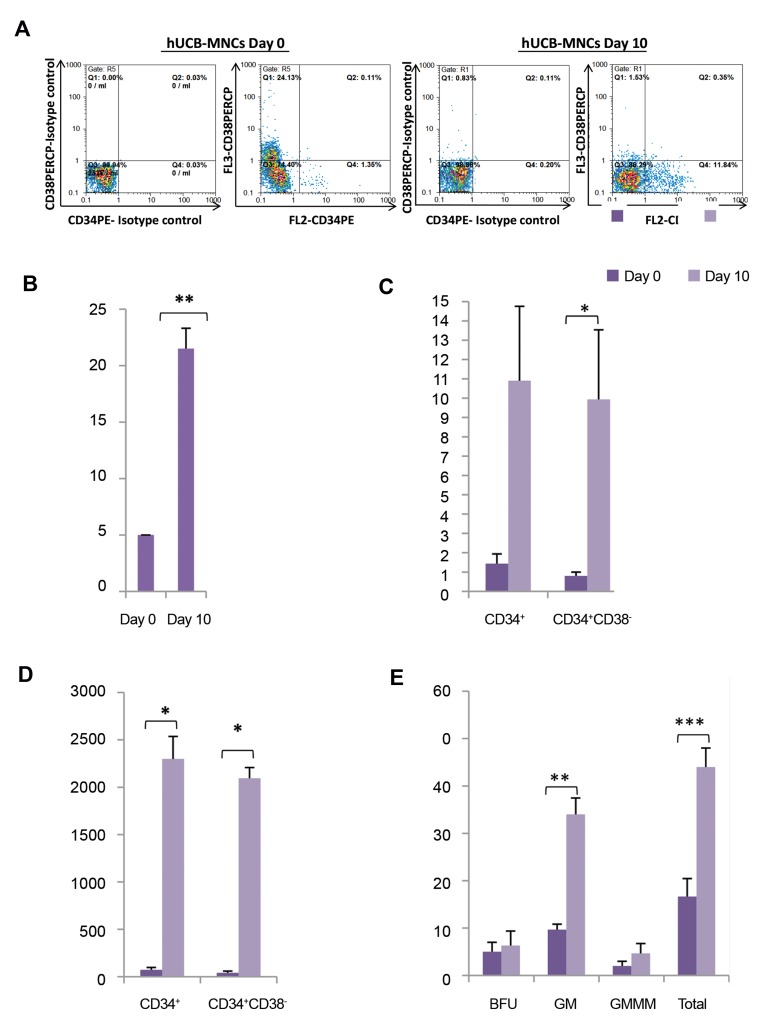
Characterization of human donor cells. **A.** Representative dot plots of hUCB-MNCs before and after expansion, **B.** Number of the total nuclear cells was significantly increased after expansion for 10 days in STF medium (n=9, **; P<0.01), **C.** Percentage of CD34^+^ and CD34^+^ CD38^-^ cells in hUCB-MNCs at day 0 and after 10 days of expansion in STF medium, **D.** Number of CD34^+^ and CD34+CD38- cells were significantly increased after 10 days expansion in STF medium (n=9, *; P<0.05), and **E.** CFU number in 2000 cells of day 0 uncultured hUCB-MNCs and the progeny of an equivalent number of expanded hUCB-MNCs (n=3, ** P<0.01, ***; P<0.001). Fold expansion was calculated by dividing the absolute output number of the expanded cells expressing a specific phenotype after 10 days of culture by the respective number on day 0. hUCB-MNCs; Human umbilical cord blood-mononuclear cells, CFU; Colony forming unit, STF; SCF+TPO+FLT3L, BFU; Burst forming unit, GM; Granulocyte-macrophage, and GEMM; Granulocyte erythrocyte macrophage monocyte.

In the first step, we sought the *in vitro* self-renewal and differentiation potential of hUCB-MNCs either before or after culture. For this purpose, freshly isolated hUCB-MNCs were cultured under very common expansion system, in serum free media containing SCF, TPO and Flt3L (STF) for 10 days. We firstly observed that number of total nucleated cells was significantly increased up to 4.3-fold after culture with STF (Fig .2). Moreover, we found that there was respectively 32 and 52.3 fold increases in the number of CD34^+^ cells and more primitive HSCs (CD34+CD38- cells).

The colony-forming rate and differentiation potential were examined by CFU-assay. As seen in Figure 2E, following the expansion, significant increases were observed in granulocytemonocyte (GM) and total CFU numbers, suggesting that the number of hematopoietic progenitor cells (HPCs) is enhanced after expansion.

### CXCR4 overexpression and increased *in vitro* homing potential of *ex vivo* expanded human umbilical cord blood-mononuclear cells

**Fig.3:**
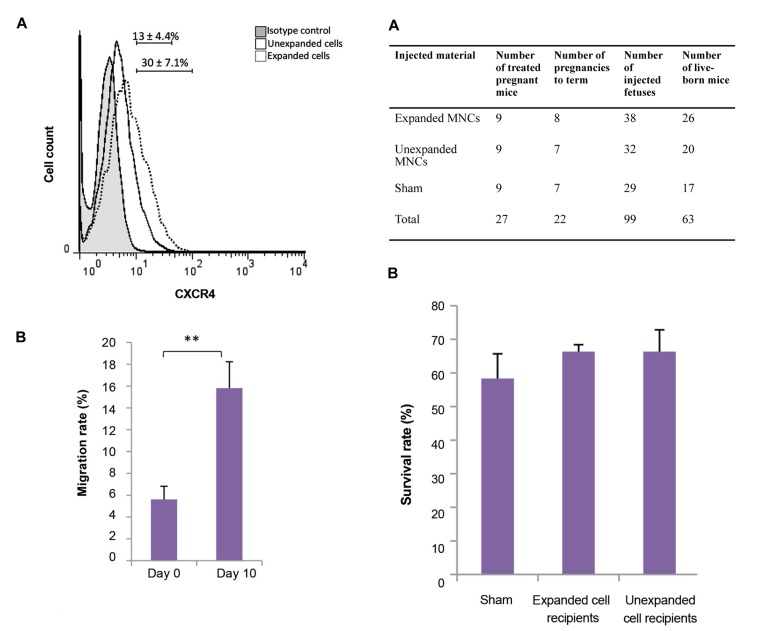
CXCR4 overexpression and increased *in vitro* homing potential of the expanded cells. **A.** Representative ﬂow-cytometer analysis of CXCR4 expression in different cells. Filled curves indicate isotype control and unfilled curves indicate labeled cells and **B.** Percentage of the STFexpanded hUBC-MNCs moved through the transwell in response to SDF- 1 versus uncultured cells (day 0) (**; P<0.01, n=5). STF; SCF+TPO+FLT3L, SDF-1; Stromal cell derived factor-1, and hUCB-MNCs; Human umbilical cord blood-mononuclear cells.

Homing and engraftment of HSCs is strictly depending on SDF-1/CXCR4 axis which can adversely be affected during in vitro culture (20). So, to determine effect of cytokine treatment on the homing ability of the expanded cells, expression of CXCR4 protein was evaluated before and after culture, using flow-cytometer. As shown in Figure 3A, the expanded cells expressed higher (2.3 fold) level of CXCR4 protein compared to the unexpanded cells. Moreover, regarding in vitro migration assay, 2.8-fold more STF-expanded cells were migrated toward the SDF-1 medium compared to the uncultured cells (Fig .3B). Therefore, it seems that hUCBMNCs culture can increase *in vitro* homing ability of the expanded cells, which might be resulted from overexpression of CXCR4 receptor.

### Outcome of in utero surgery

**Fig.4:**
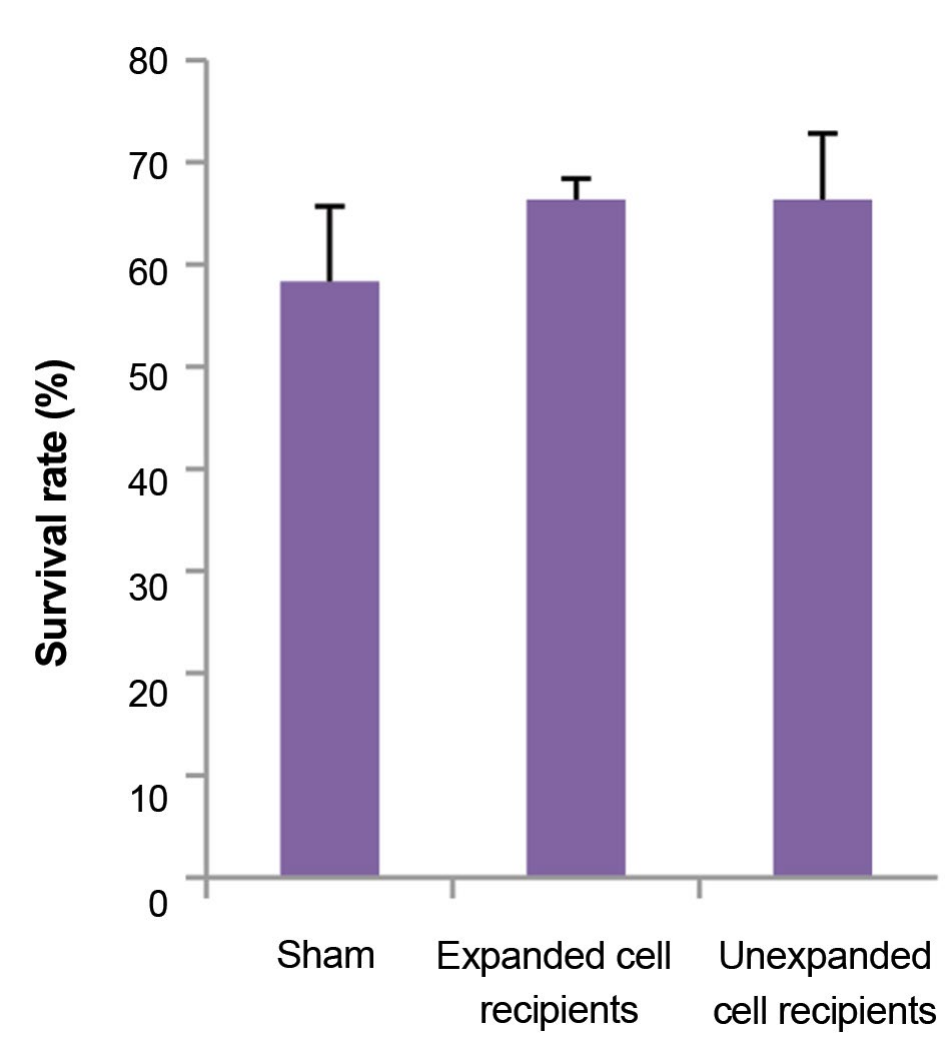
Outcome of in utero surgery. A. Number of the pregnant mice and their embryos used through the whole experiment and B. Comparison of survival rates within expanded cell-recipients, unexpanded cell-recipients and sham. MNC; Mononuclear cells.

Figure 4A shows the surgical outcomes of 4 independent experiments. At 11.5-13.5 days of gestation, 21 pregnant mice were under surgery, out of which 5 mothers died due to surgical complications such as bleeding and prolonged anesthesia.

Totally, from mothers surviving surgery, 99 transplanted fetuses were under surgery, among which STF expanded cells, unexpanded cells and PBS were injected to respectively 38, 32 and 29 fetuses. In overall, the live birth rate was 63.6%. As seen in Figure 4B, recipients show similar viability injected by either STF expanded MNCs (68.4%) or unexpanded MNCs (62.5%). Furthermore, all of the live-born fetuses were normal and had no sign of malformations.

### Short-term *in vivo* homing of human umbilical cord blood-mononuclear cells in liver and spleen of recipients

**Fig.5:**
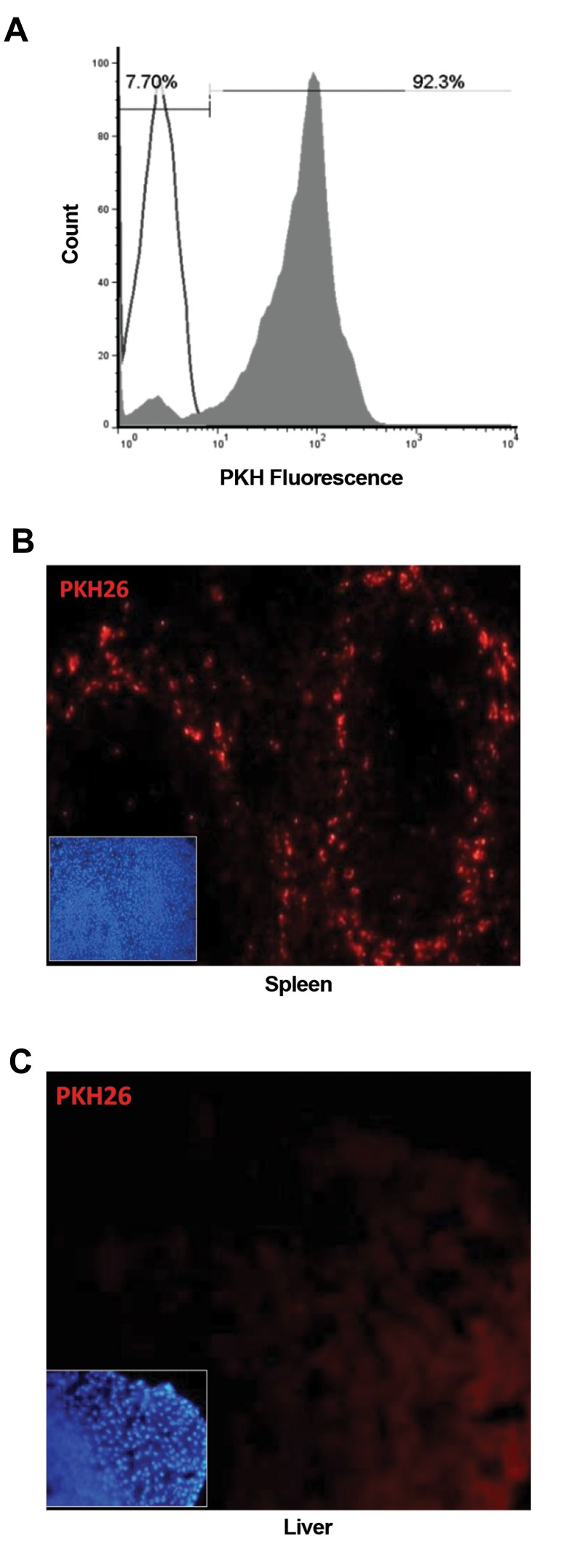
Short-term *in vivo* homing of hUCB-MNCs. **A.** Analysis of PKH26 fluorescence of 20000 MNCs by flow-cytometer, before (unfilled curve) and after staining with PKH26 (gray filled curve) and **B.** Identification of PKH-stained hUCB-MNCs, 48 hours after infusion. Prepared frozen sections from the spleen and liver of transplanted fetuses were screened for the presence of PKH-bright cells (red: PKH26, blue: DAPI). hUCB-MNCs; Human umbilical cord blood-mononuclear cells.

At first, transplanted fetuses were analyzed for presence of the injected cells. For this purpose, freshly isolated MNCs were labeled with a viable fluorescent membrane dye PKH26 to track after infusion (Fig .5A). Around 15-16 days of gestation, liver and spleen organs are the hematopoietic sources in the fetal mouse (21). Therefore, one set of fetuses were sacrificed 48 hours after transplantation and PKH-positive cells were tracked in the frozen sections prepared from the mentioned tissues. As shown in Figure 5B, the highest level of homing was found in spleen, while some PKH-positive cells were also detected in liver.

### Expanded human umbilical cord blood-mononuclear cells have higher engraftment potential than unexpanded cells

**Fig.6:**
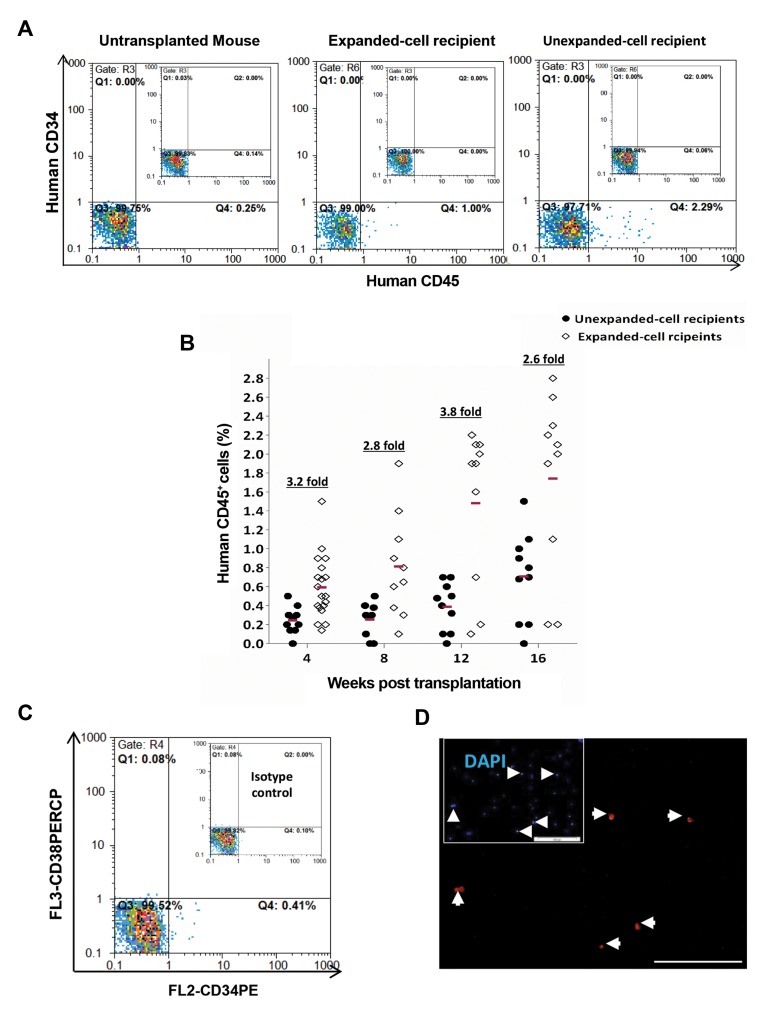
The *ex vivo* expanded hUCB-MNCs have higher engraftment potential than unexpanded cells. **A.** Representative flow-cytometer analysis for human cell engraftment in peripheral blood of the expanded- and unexpanded-cell recipients. Peripheral blood of normal mouse was employed as negative control, **B.** Mean human engraftment levels in peripheral blood of NMRI mice fetal transplanted with expanded or unexpanded hUCBMNCs. Mice with ≥0.2% human cells were considered chimeric, **C.** Representative flow-cytometer analysis for human cell engraftment in peripheral blood of the expanded- and unexpanded-cell recipients, and D. Identification of human CD45^+^ cells in the bone marrow of recipient mice. Bone marrow smears of 4-months-old transplanted mice were screened for the expression of human nuclear antigen (arrows; As mentioned they are human CD45<sup>+</sup> cells). hUCB-MNCs; Human umbilical cord blood-mononuclear cells.

To evaluate the engraftment, flow-cytometry analysis of the human CD45 marker was performed for the first time in peripheral blood of 3 weeks old mice (4 weeks after transplantation). To reliably eliminate background signal, the isotype control antibody was recruited. Peripheral blood cells from a normal mouse were also analyzed as an additional control for this analysis method (Fig .6A). Newborn mice were considered to be chimeric, if ≥ 0.2% CD45^+^ human cells were present in their peripheral blood sample.

Totally, CD45^+^ human cells were detected in 73% (19/26) of live born mice that injected with the expanded MNCs, while only 35% (7/20) of unexpanded-cell recipients had become chimera. Previous studies have shown an increased engraftment level of human HSCs in sheep and mice, following treatment with human hematopoietic growth factors (hHGFs). Therefore, newborn recipients were treated with subcutaneous injections of different HGFs (IL3, SCF and G-CSF), 3 times a week beginning at 4 weeks after transplantation. As demonstrated in Figure 6B, at the beginning, expanded-cell recipients displayed a higher level (1.8-fold) of human engraftment compared to the unexpanded-cell recipients, while it was dramatically amplified after treatment by hHGFs. Precisely, compared to the other group, expanded-cell recipients showed 3.2-, 3.8- and 2.6-fold increases in engraftment at 8, 12 and 16 weeks after transplantation, respectively. As shown in Figure 6C, human originality and functionality of the transplanted cells were additionally confirmed by the presence of anti-HNA in the bone marrow cells of 4 months old mice treated with hHGFs. Interestingly, there was a downward trend in the engraftment of recipients that did not treat with hHGFs and injected by either expanded or unexpanded cells. So that, after 4 months, no human cell was seen in their blood (data not shown).

## Discussion

In this study, overall frequency of the human donor cell engraftment in NMRI recipient mice as early as 4 weeks post transplantation was <3%. 7/20 (35%) recipients of unexpanded MNCs and 19/26 (73%) recipients of expanded MNCs were chimeric. This result indicates that hUCB-MNCs expansion produces a higher level of engraftment than freshly isolated cells. Furthermore, the average level of human cells in unexpanded-cell recipients was 0.3%, while it reached to 0.55% in expanded-cell recipients. Here, the level of human cells engrafted into NMRI mice is substantially higher than the previous reports in nondefective rodents (22-25). On the other hand, IUT of human fetal liver-MNCs or fetal BM-CD34^+^ cells into NOD/SCID mice resulted in 15% expression of human cells in 10-12% of 8 weeks old mice (26). Similar to our finding, it was previously reported that *ex vivo* expanded UCB-HSCs have higher engraftment ability in IUT model of sheep: 8.1% for expanded cells versus 0.1% for unexpanded cells (27). The engraftment of human CB-derived stem cells has also been evaluated in ovine fetuses (28). In this study, only 18% of lambs, IUT hUCB-CD34^+^ cells showed human cell expression up to 0.8%. In other larger species like canine model, it has been reported that IUT of 108 haploidentical CD34^+^ cells/kg of fetuses resulted in <1% microchimerism (29). Different rate of the chimerism as well as the level of engraftment could be related to the route of transplantation (30), quantity and quality of injected cells (31), different isolation techniques (32), different source of HSCs (33), gestational day of injection (34) and the animal species (35). The used cytokines can also affect the chimera formation (36).

Here, we used unpurified MNCs as a cell source, since unpurified MNCs contain more primitive progenitors as well as mature cells that compete for homing space with purified CD34^+^ cells (31). On top of that, we performed IUT on E11.5-E13.5, when the highest degree of chimerism was reported (37). Although treatment by hHGFs led to higher level of engraftment in both groups, the STF-expanded MNCs were always maintained higher through weeks post transplantation. The higher engraftment potential of expanded MNCs might be due to overexpression of the homing gene, CXCR4, following expansion. Moreover, *ex vivo* expansion provides us with higher number of progenitor and mature cells including neutrophils which can engraft more rapidly, in comparison with unexpanded CD34^+^ cells (38).

In our experiments, in the absence of treatment with hHGFs, regardless of the fact those samples were subjected or not to *ex vivo* expansion, the human cells continued to decrease until they were undetectable in the host body. This indicates that expanded cells lack the ability to longterm engraftment. This data also highlights the importance of compatibility between the hematopoietic environment of donor cells and the host body.

## Conclusion

Here, we successfully demonstrated application of mouse IUT model to assess engraftment potential of hUCBMNCs. Although the IUT model allows transplantation of xenogeneic cells without host conditioning, the frequency and levels of donor cells are significantly low. These data support the idea that despite the immaturity of fetus’ immune system, there are some barriers preventing the engraftment of human cells. It seems that mainly overcoming the conflicts of hematopoietic environment as well as attenuating the immune response against the donor cells will make IUT model as an acceptable model for basic and pre-clinical research.
